# Fabrication of fluorescent pH-responsive protein–textile composites

**DOI:** 10.1038/s41598-020-70079-x

**Published:** 2020-08-03

**Authors:** Dalia Jane Saldanha, Zahra Abdali, Daniel Modafferi, Bita Janfeshan, Noémie-Manuelle Dorval Courchesne

**Affiliations:** Department of Chemical Engineering, McGill University, 3610 University Street, Montreal, QC H3A 0C5 Canada

**Keywords:** Biomaterials - proteins, Biosensors

## Abstract

Wearable pH sensors are useful tools in the healthcare and fitness industries, allowing consumers to access information related to their health in a convenient manner via the monitoring of body fluids. In this work, we tailored novel protein–textile composites to fluorescently respond to changing pH. To do so, we used amyloid curli fibers, a key component in the extracellular matrix of *Escherichia coli*, as genetic scaffold to fuse a pH-responsive fluorescent protein, pHuji. Engineered amyloids form macroscopic and environmentally resistant aggregates that we isolated to use as stand-alone hydrogel-based sensors, and that we trapped within textile matrices to create responsive bio-composites. We showed that these composites were mechanically robust and vapor-permeable, thus exhibiting favorable characteristics for wearable platforms. CsgA–pHuji fibers integrated in the textile allowed the final device to respond to pH changes and distinguish between alkaline and acidic solutions. We demonstrated that the resulting composites could sustain their fluorescence response over days, and that their sensing ability was reversible for at least 10 high/low pH cycles, highlighting their potential for continuous monitoring. Overall, we introduced a biosynthesized amyloid-based textile composite that could be used as biosensing patch for a variety of applications in the smart textile industry.

## Introduction

Epidermal pH is an effective biomarker for preliminary detection of skin-related ailments such as dermatitis, acne and other bacterial and fungal infections^1^. The pH of healthy skin is slightly acidic (pH 4–6.5) due to the presence of an ‘acid mantle’, a protective barrier against harsh environmental stresses and invasive organisms^2^. Low epidermal pH also promotes vital physiological processes in the skin, including but not limited to, antimicrobial defense, periodic skin differentiation and shedding^3^. Monitoring changes in epidermal pH is therefore an essential aspect of skincare and dermatological diagnostics.

Wearable sensors that can be directly mounted onto one’s body to detect and respond to the physiological status of the wearer’s skin are making diagnostic data more accessible^4^. Irrespective of their final application, wearable devices are ideally designed to be flexible (in order to blend seamlessly with movements of the body), tunable (to detect diverse physiological signals) and biocompatible^5^. However, most platforms for pH sensing currently employ electrodes or organic dyes to detect pH electrochemically or optically^6–9^. Using electrodes limits the mechanical flexibility of the system, renders the device sensitive to background motion, and often requires multistep chemical functionalization processes^10^. Dyes used in wearable sensors face a major issue of not being easily tunable—requiring complex chemical modifications to detect and bind new analytes. To prevent leaching out from sensors during use, devices often incorporate rigid substrates that are less likely to disintegrate in contact with solvents or diverse chemical environments. With the exception of soft elastomers and textiles that are now being increasingly used for wearable chemical sensing, the rigidity of most substrates restrains the movement of sensors on the body^11^. Nonetheless, recent developments in the field of protein-based materials have enabled the fabrication of macro-sized films, patches and coatings that can be both mechanically flexible and responsive^12–16^. Protein-based materials are genetically customizable, which allows for the introduction of on-demand sensing domains, and they are readily biocompatible, making them attractive materials to fabricate wearable sensors^17^.

Here, we report the fabrication of textile–protein composites as flexible, easily customizable, and bioresponsive candidates for wearable fluorescent biosensors. For this application, we chose curli fibers, polymers of repeating CsgA proteins produced by *Escherichia coli* (*E. coli*) bacteria, as an adaptable matrix for the sensor. CsgA can be engineered to incorporate large and complex insertions at its C-terminal. These insertions can be diverse in origin and application, including but not limited to, trefoil factors for gut healing, metal binding domains for surface adherence and fimbrial adhesins for vaccines against urinary tract infections^13,18–20^. These fusion proteins can then self-assemble in bacterial growth media to form amyloid curli fibers that are innately resistant to harsh chemicals and proteases naturally present in sweat^21^. Engineered curli fibers can also be used to create macroscopic thin films, hydrogels and coatings^12,13,22,23^. To diversify the range of their applications, amyloid-based biomaterials can be integrated with various suitable substrates. To date, curli fibers have been used as a composite with polysaccharides (e.g. alginate), ceramics, polystyrene, mussel foot protein and textiles (cotton) to create systems that are mechanically stable and genetically versatile^14,24–26^. Most composites fabricated so far, however, are small (~ 1 cm^2^), and are limited by yield of curli fibers obtained after purification or by the efficiency of the deposition methods of bacterial biofilms onto substrates. Combining curli fibers with larger substrates has therefore come at the cost of achieving a low protein loading within the system, as exemplified by a recent light-inducible biofilm-fabric composite in which curli fibers were uniformly distributed but did not fully cover the fabric^24^. In addition, some of the integration techniques for proteins in textiles involve electrospinning, a technique requiring toxic solvents (e.g. chloroform), calling for safer manufacturing protocols^27^.

To fabricate composite materials with a high surface coverage, in a simple and rapid fashion, we have adapted a scalable vacuum filtration process to integrate curli fibers with textiles, and have applied this process to a pH-responsive curli fiber fusion^12^. Rather than employing vacuum filtration to purify and isolate curli fibers from *E. coli* cells as previously described^12^, we have utilized vacuum to force curli fibers into porous textiles and uniformly trap the fibers to form functional composites.

Specifically, we present a fluorescent curli fiber-based sensor created by genetic fusion of CsgA with pHuji, a pH-responsive variant of the red fluorescent protein mApple^28^.

Upon excitation at 568 nm, pHuji fluoresces at 598 nm under alkaline conditions with a pKa of 7.8^28^. Since most clinical and cosmetic concerns of the skin (e.g. sunburns, allergies and fine lines) are associated with higher skin pHs^29^, pHuji was considered to be an apt candidate for epidermal pH detection. We have characterized the CsgA–pHuji fusion as a responsive protein hydrogel, which also retained its properties after entrapment in nonwoven textiles like acrylic. We have studied the response of our composites to varying mechanical and environmental stresses and also observed that it remained sensitive to pH for prolonged periods of time. Further, we evaluated the reversibility of the textile–protein sensor, which renders it a promising candidate for continuous monitoring applications in healthcare. Through this work, we aimed to establish composites of textiles and engineered curli fibers as versatile and biofriendly biosensing fabrics for detection of pH, and eventually of other analytes of physiological significance.

## Results

### Integration of curli fibers in textiles via vacuum filtration

**Figure 1:**
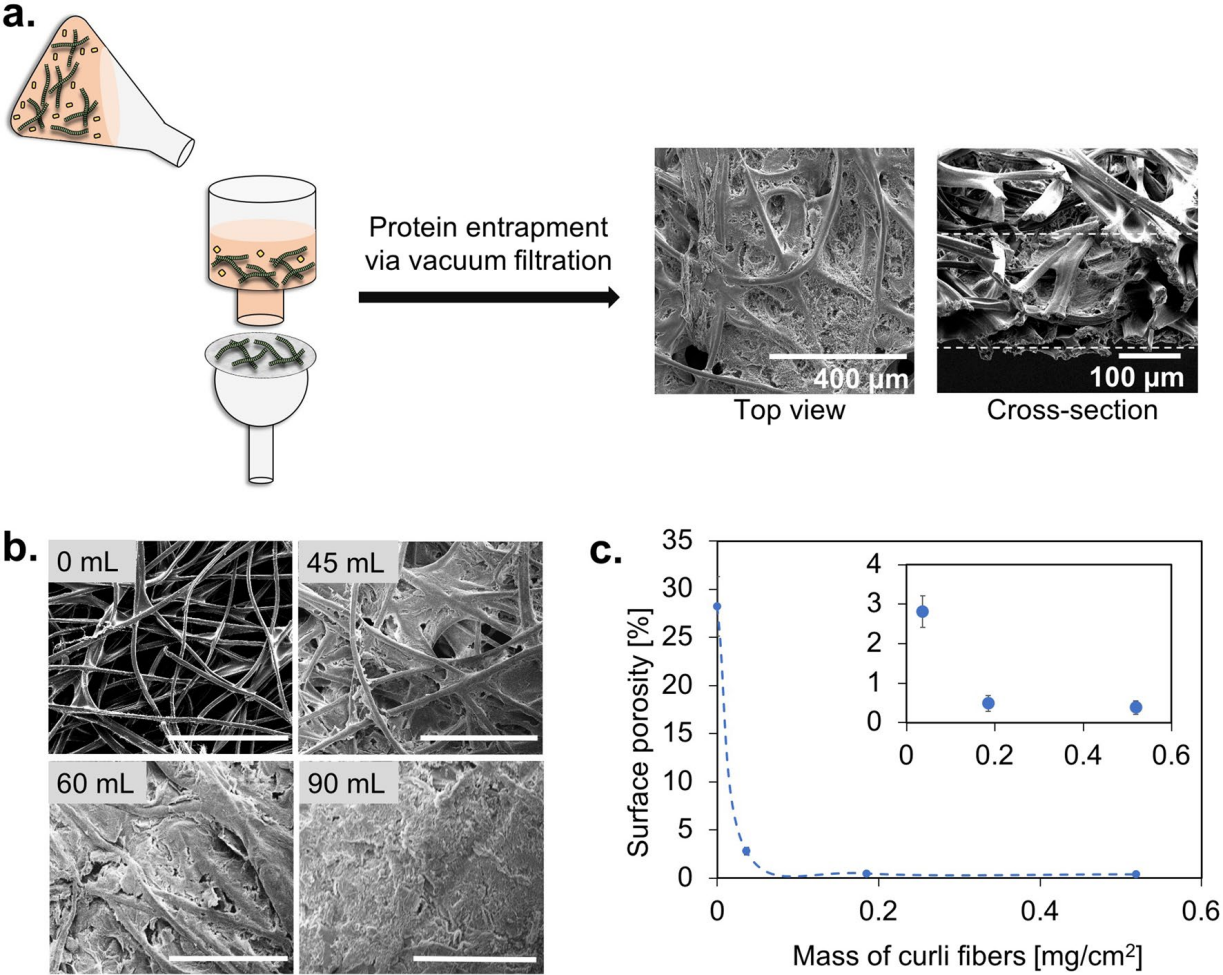
Integration of curli fibers with textiles via vacuum filtration. (**a**) A scheme of the vacuum filtration protocol adapted for textile–protein composite fabrication. (**b**) SEM images of textile–protein composites created using 0, 45, 60 and 90 mL of curli producing bacterial cultures. The protein content in the composites are 63 mg/cm^2^, 185 mg/cm^2^ and 500 mg/cm^2^ for 45, 60 and 90 mL of culture filtered, respectively. Scale bars: 300 µm. (**c**) Surface porosities of the different composites and acrylic control analyzed using ImageJ. Inset: Surface porosities of acrylic–curli composites only, excluding the acrylic control with high porosity.

To develop wearable diagnostic platforms from curli fibers, we first designed a protocol to fill the pores of textiles with protein materials, using wild type (WT) curli fibers as model proteins. Vacuum filtration had previously been used to isolate curli fiber hydrogels from bacterial cultures, with a protocol consisting of an incubation with a denaturing agent, guanidinium hydrochloride (GdmCl) to lyse bacterial cells; an incubation with benzonase nuclease to remove nucleic acids; and a final incubation in a surfactant, sodium dodecyl sulfate (SDS), to facilitate delamination and also enhance gelation of the protein product; all of which were followed with water rinses^12,13^. Here, to trap fibers in the textiles rather than isolate and collect them, we eliminated the final incubation in SDS and collected the textile-composite material as our product (Fig. 1a).

We then identified textiles that would effectively serves as porous substrates for the integration of protein-based polymers via vacuum filtration. To do so, we chose candidates that were currently in extensive use in the clothing industry and possessed industrial value such as cellulose acetate, polyvinylidene fluoride, silver coated nylon, nylon-6, polyvinyl rubber, polyvinyl butyral, polyacrylonitrile and non-woven acrylic (see Supplementary Fig. S2a). We compared the selected textiles on the basis of their pore size and mechanical strength. In order to employ vacuum filtration and allow bacteria to pass through the pores of the textiles but not aggregated curli fibers (which measure tens to hundreds of microns in size), the pore size of the textiles had to be greater than 5–10 microns and smaller than ~ 100 microns. Using SEM, we observed greater bacterial contamination on fabrics with pore sizes below 10 µm^2^, and minimal bacterial trapping for non-woven acrylic textiles with larger pores (500 µm^2^) (see Supplementary Fig. S2b). We further tested for structural integrity of the textiles after being subjected to the vacuum filtration-based curli trapping protocol. Materials like polyvinyl rubber, polyacrylonitrile, polyvinyl butyral, cellulose acetate and polyvinylidene fluoride broke and/or permanently deformed during the process (see Supplementary Fig. S2c,d). Hence, we chose to develop our proof-of-concept textile–protein composites using non-woven acrylic.

Vacuum filtration of curli expressing bacterial cultures on acrylic yielded a uniform protein loading throughout the textile matrix. SEM images of textiles after filtration showed dense and uniform packing of the pores with curli fibers, both from the surface of the textile and throughout the cross-section (Fig. 1a). To optimize the textile–protein composite fabrication process, we filtered different volumes of WT CsgA culture ranging from 45 to 90 mL through 17.4 cm^2^ circular textile samples. We weighed the textiles before and after protein integration to determine the mass of protein incorporated, which ranged from 0.03 mg cm^−2^ for 45 mL of culture to 0.5 mg cm^−2^ for 90 mL of culture. Using SEM, we observed the surface of the samples and observed an increasing pore filling fraction with an increasing volume filtered. Filtering at least 60 mL of culture allowed to completely fill the top surface pores, while larger volumes contributed to creating layers on the surface of the textiles and increased bacterial trapping (Fig. 1b). 60 mL of culture allowed us to trap 0.185 mg of curli fibers per cm^2^, resulting in a low surface porosity of 2.8% (Fig. 1c). We therefore continued our subsequent investigations with a fixed volume of culture of 60 mL.

### Mechanical and physical characterization of acrylic–curli composites

**Figure 2:**
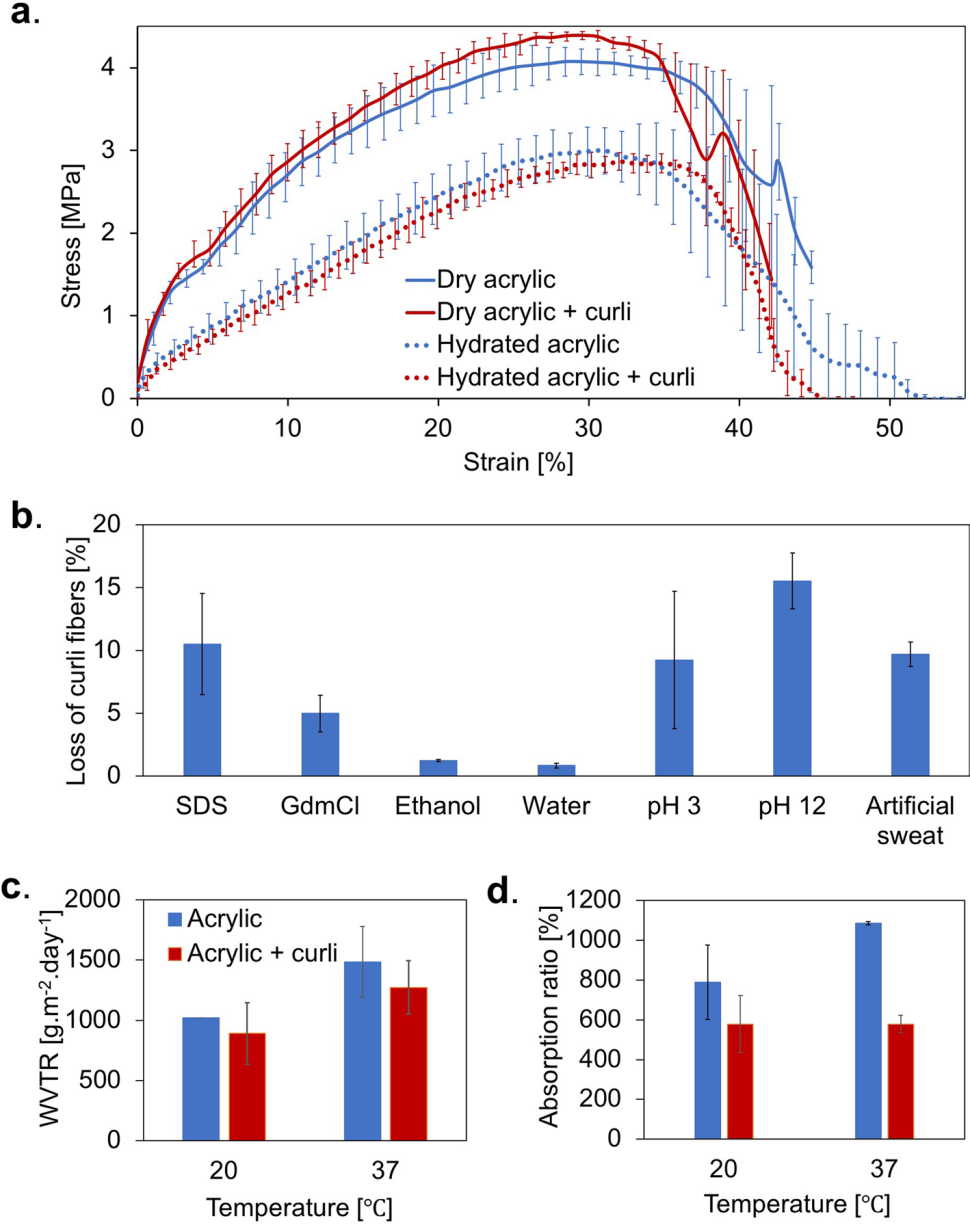
Mechanical and physical characterization of acrylic–curli composites. (**a**) Stress–strain curves for acrylic and acrylic–curli composites in the dry and hydrated states. (**b**) Percentage loss of curli fibers from acrylic–curli composites in the presence of various chemical reagents over 10 days. (**c**) Water vapor transmission rates (WVTR) of acrylic and acrylic–curli composites at room temperature and human body temperature. (**d**) Water absorption ratios of acrylic and acrylic–curli composites at room temperature and human body temperature.

We then tested the composites for properties of interest for wearable sensor applications: changes in mechanical properties upon incorporation of protein, moisture control (to prevent leakage of biofluid), gas permeability (to allow aeration of contact skin), and resistance to environmental stresses (detergent action, salts and other reagents involved in manufacture). First, to test their tensile strengths, we cut textile–curli fiber composite strips of 60 × 10 mm with an average fiber loading of 0.65–0.85 mg cm^−2^, and we performed elongation tests to determine their breakage points, both in the dried and hydrated states. We compared the composites with plain acrylic strips as controls. Dry non-woven acrylic is a ductile material that can stretch to almost 45% its original length before rupture, a property the acrylic–curli composite retained, as seen in Fig. 2a. In the hydrated state, the composites and controls exhibited similar ranges of ductility**,** but at the cost of a slight decrease in tensile strength compared with the dry samples (~ 4 MPa dry, and ~ 3 MPa wet). The mechanical analyses established that the vacuum filtration protocol and integration with curli fibers did not adversely impact the mechanical properties of acrylic.

We then tested the stability of the curli fibers on the textile matrices against the various environmental stresses that they are likely to be subjected to during manufacture and/or use as sweat sensors. We incubated 1 cm^2^ acrylic–curli composite samples with a denaturing agent (GdmCl), a detergent (SDS), solvents (water and ethanol), buffers with varying pHs (pH 4 and pH 12), and artificial sweat, for 10 days. Our analysis of the mass of curli fibers trapped in the textiles before and after incubation (Fig. 2b) revealed that solvents like water and ethanol caused minimal loss of protein during incubation (0.8–1.25%). The presence of salts in the surrounding medium, however, resulted in increased loss of protein from the composite, alkaline pH buffers contributing to a greater loss (15%) compared with other buffers (9–10%). However, even after agitation at 50 rpm and continuous incubation of curli-acrylic composites with potentially destabilizing reagents, the fabricated samples still retained more than 85% of their original protein content. The retention of protein fibers within the textile matrix indicates the effectiveness of the curli fiber entrapment protocol and its potential ability to survive chemical treatments when used for periods as long as 10 days.

We studied additional properties of the curli-acrylic composite including its water absorption ratio and water-vapor transmission rate. To obtain an estimate of the breathability of our composite, we calculated the water vapor transmission rate of acrylic–curli composites and found it to be between 891–1273 g m^−2^ day^−1^. This rate falls in the range of many synthetic epidermal sensors reported thus far, one of the factors that allows us to establish its compatibility with skin (with a WVTR of 200–600 g m^−2^ day^−1^)^30–32^. Also, water vapor transmission was higher at temperatures closer to body temperature (37 °C), a phenomenon possibly occurring due to the expansion of the matrix at higher temperatures, thereby promoting gas diffusion (Fig. 2c). Acrylic can swell to almost 800–1000% of its original dimensions, depending upon the ambient temperature. The incorporation of curli fibers into this matrix reduced the swelling ratio to a constant 600% at both temperatures tested. However, the water absorption of the composite was still in the range of most commercial skin dressings (200–1000%) and the ability for dried curli fibers in the pores to reconstitute into hydrogels in the presence of water allowed the textile to maintain a constant swelling ratio across the temperatures tested^33,34^ (Fig. 2d).

### Biosynthesis of CsgA–pHuji fibers

**Figure 3:**
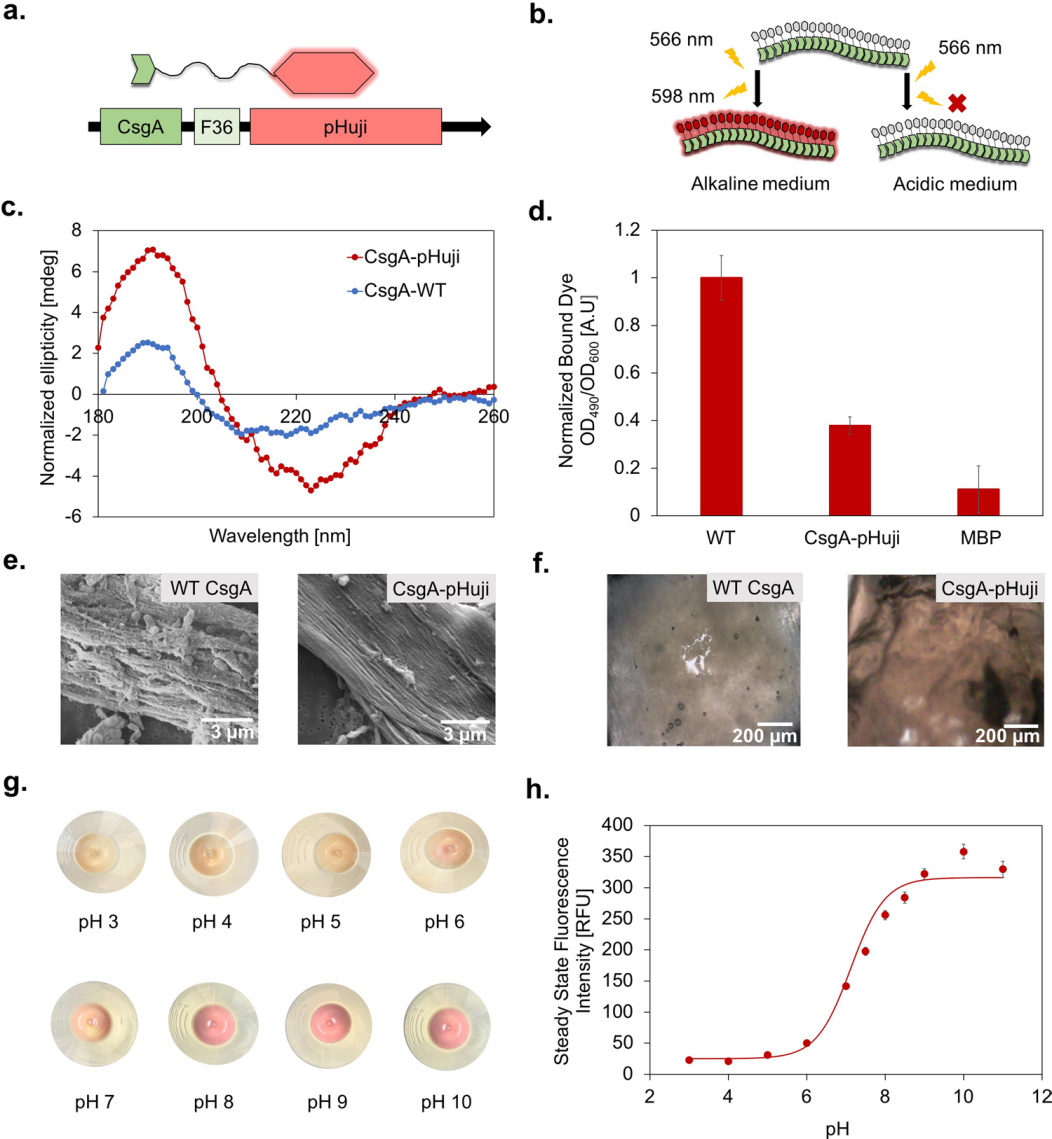
Genetic engineering and expression of responsive fluorescent curli fibers. (**a**) A scheme representing fusion of CsgA with a flexible linker and pHuji. (**b**) The fluorescence response of CsgA–pHuji under different pH conditions. (**c**) Circular dichroism spectra normalized for concentration of WT CsgA and CsgA–pHuji. (**d**) Normalized amount of dye bound to pellets of WT CsgA and CsgA–pHuji bacterial cultures via a Congo Red pull-down assay. (**e**) Optical microscope images of freshly purified WT CsgA and CsgA–pHuji hydrogels. (**f**) SEM images of WT CsgA and CsgA–pHuji producing bacterial cultures. (**g**) pH assays with concentrated pellets of CsgA–pHuji producing biofilms. (**h**) Mean steady state fluorescence response of CsgA–pHuji hydrogels to different pH buffers ranging from 3–11. The non-linear fit was performed using the Henderson–Hasselbalch equation (Eq. 4).

To produce a pH-responsive curli hydrogel, we genetically fused pHuji to CsgA along with a flexible linker (Fig. 3a). We expressed CsgA–pHuji fibers and isolated them via vacuum filtration, using a previously established protocol that allows for the spontaneous formation of curli fiber hydrogels. We analyzed the composition of the semi-purified protein hydrogels through SDS-PAGE (see Supplementary Fig. S1). To determine whether CsgA and pHuji can fold into their native structures when expressed as fusion proteins, we studied the secondary structures of CsgA and CsgA–pHuji using circular dichroism (CD) (Fig. 3c) on the isolated protein hydrogels. As expected, we observed a positive peak around 195 nm and negative band around 210–220 nm for WT CsgA, characteristics of the five anti-parallel β-sheets composing a curli subunit^35^. We also observed two peaks for CsgA–pHuji. The positive peak at 195 nm was of greater intensity compared with WT CsgA, indicating a greater β-sheets content in the fusion protein. The negative peak was also more intense compared with WT CsgA, and it was slightly shifted towards 222 nm. This shift could reveal the contribution of α-helices to the structure. In fact, while the structure of pHuji has not been elucidated yet, its structural similarities with a red fluorescent protein, mCherry, have been confirmed^28^. mCherry (PDB ID: 2H5Q) possesses 13 β-sheets and 3 α-helices forming a β-barrel structure, consistent with our CD observations. Circular dichroism thus points towards the fact that both CsgA and pHuji have normal secondary folds, sustained throughout the expression and purification processes.

Next, to confirm whether CsgA–pHuji fusion proteins could form amyloid aggregates characteristic of curli fibers, we assayed the bacterial-curli pellets with the Congo Red dye^36^. Congo Red has an affinity for amyloids that allows us to detect the presence of curli fibers in concentrated cultures. As seen in Fig. 3d, the pellet for the culture producing WT CsgA bound more Congo Red molecules compared with CsgA–pHuji, indicating an overall lower yield of formation of amyloid fibers for the fusion protein. This difference could be a result of impaired secretion of CsgA–pHuji outside the cells or to changes in polymerization patterns of CsgA in the presence of pHuji, both hypotheses could be due to the bulky structure of pHuji and to folding differences. Nevertheless, the yield of CsgA–pHuji hydrogels obtained after vacuum filtration was sufficient to proceed with subsequent macroscale applications.

To visually ascertain the formation of amyloid fibers for CsgA–pHuji at the microstructural level, we also imaged samples from the two bacterial cultures using SEM (Fig. 3e). For both WT CsgA and CsgA–pHuji, we observed aggregated and elongated fiber-like structures characteristic of curli fibers expressed in the absence of the CsgB nucleator protein^12^.

### Analysis of pH-responsive behavior of CsgA–pHuji proteins

As a first step towards establishing the pH-responsiveness of CsgA–pHuji hydrogels, we first simply observed color differences between the WT CsgA and CsgA–pHuji hydrogels and bacterial pellets. CsgA–pHuji hydrogels showed a red tint characteristic of pHuji when exposed to a neutral to alkaline pH (Fig. 3f). We performed a study to visualize color changes of pelleted bacterial cultures incubated in buffers of varying pHs. The change in color of the pellets was visible to the naked eye, with the red color appearing above pH 7 (Fig. 3g). Next, to quantify this response, we incubated freshly purified CsgA–pHuji hydrogels in standard Carmody buffers from pH 3 to 11 for 1 h and measured their fluorescence emission. The fusion proteins, upon excitation at 550 nm, exhibited negligible fluorescence at 598 nm between pH 3–6. The fluorescence began to increase above pH 6 until it reached a stable level at around pH 9 (Fig. 3h). This sigmoidal behavior is consistent with previously reported curves for pure pHuji proteins^28^, indicating that the core fluorophore of pHuji remains functional as a fusion with CsgA and after being exposed to chemical treatments during vacuum filtration. There are minor differences between our fusion construct and the wild type pHuji reported in literature, namely a shift in pKa (7.1 and 7.7, respectively) and the fluorescence fold-changes they exhibit between pH 5.5. and 7.5 (a 7- and 20-fold increase, for our fusion and WT pHuji respectively)^28^. These differences are, most likely, a result of minor shifts in the environment surrounding CsgA–pHuji’s core fluorophore. The study of the response of the hydrogel over a 24-h period allowed us to also evaluate the kinetics of the response. We observed that it took 1 h for the fluorescence to reach steady state values at every pH, and that fluorescence was then maintained over at least 1 day (see Supplementary Fig. S4). However, even after 5 min, we detected the same sigmoidal trend as a function of pH, which was maintained at every time point.

### Fabrication of pH-responsive sensors on textiles

Based on the robust fluorescence response of CsgA–pHuji at pHs relevant for diagnosing skin problems, we proceeded with the integration of the fusion protein with textile scaffolds to fabricate wearable sensors. The composite of CsgA–pHuji and acrylic exhibited similar patterns of pore filling and protein distribution at the microstructural level as the WT composite after vacuum filtration of 150 mL of the CsgA–pHuji culture (see Supplementary Fig. S3). The mechanical behavior of the sensor containing the engineered CsgA fusion also followed the same trend as for acrylic–WT CsgA composites, with similar tensile strength in the dried state (Supplementary Fig. S3).

**Figure 4:**
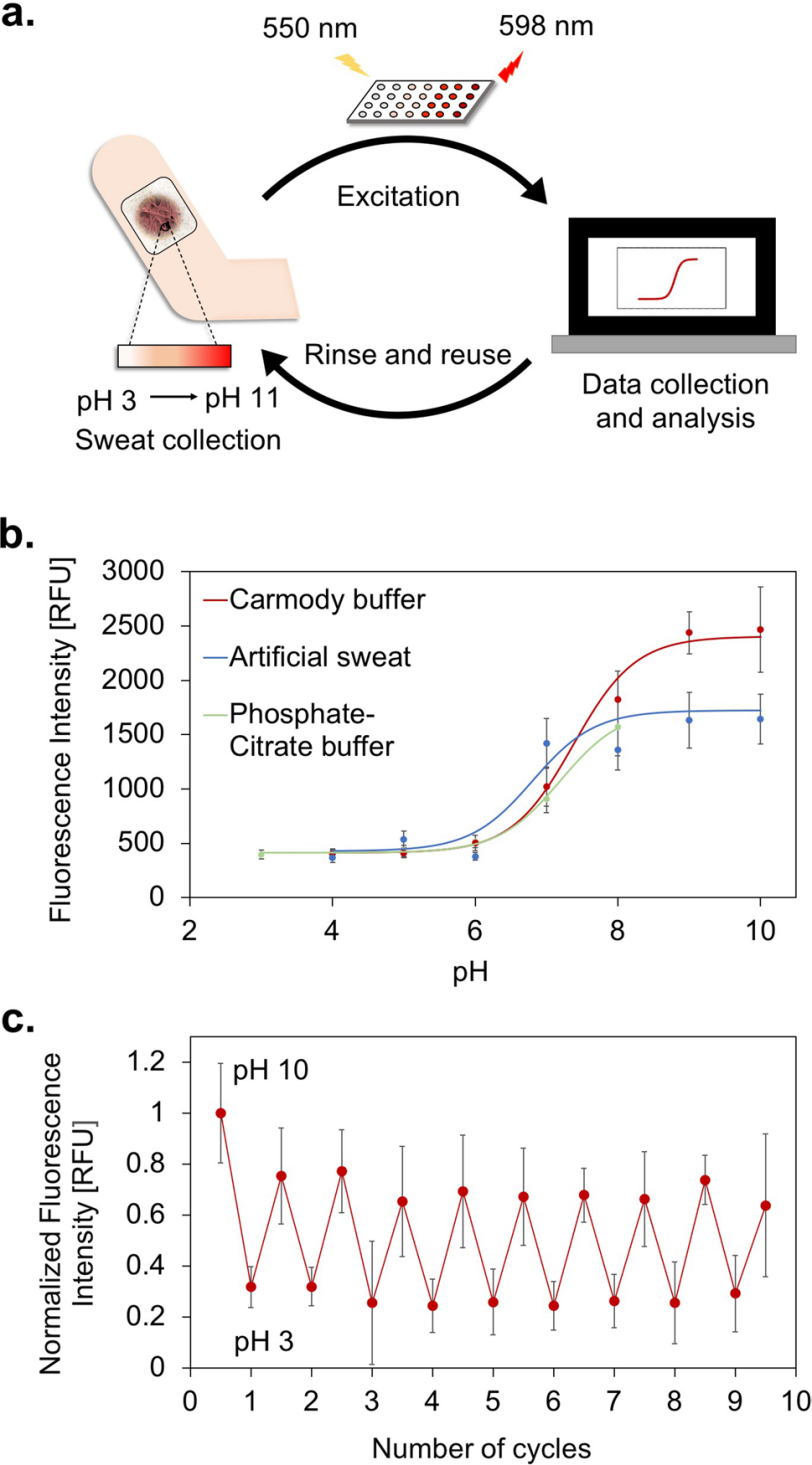
Wearable protein–textile composites for fluorescent pH sensing. (**a**) A scheme representing the steps involved in the working of our textile–protein sensor. (**b**) Fluorescence intensities of the textile at pH conditions ranging from 3–11 in three types of solutions—artificial sweat, Carmody buffers and phosphate-citrate buffers. The non-linear fit for all three curves was performed using the Henderson–Hasselbalch equation (Eq. 4). (**c**) The reversible fluorescence behavior of the acrylic–CsgA–pHuji composite observed at two distinct pHs (pH 3 and pH 10) over 10 cycles.

The use of these wearable pH sensors involves four steps: (1) interaction of the composite patch with sweat (the analyte), (2) removal of the patch and measurement of its fluorescence using a microplate reader, (3) analysis of the data compared with a calibration curve, and (4) rinse and reuse of the sensor (Fig. 4a). We quantified the sensor’s response by measuring its fluorescence. We plotted calibration curves for our sensor’s fluorescence response with Carmody buffers (between pH 4–10), phosphate-citrate buffers (between pH 3–8) and artificial sweat (between pH 4–10). The results revealed the same sigmoidal trend for CsgA–pHuji trapped within acrylic (for all three buffers) and the one seen for the hydrogel (Fig. 4b). The pKa values of the three calibration curves varied between 6.8 in artificial sweat to 7.4 in the buffers and are very close to what is reported for pure pHuji in literature (7.7)^28^. The similarity of our system’s pKa values to that in literature implies that the dielectric environment surrounding the fluorophore is not compromised in the fusion protein (both in the hydrogel state and after integration with the textile). The pKa value of purified pHuji has been retained in the engineered construct, indicating a conservation of structure and folding patterns, as discussed before^28^. We were also able to observe subtle changes in the color of the textile– CsgA–pHuji composite under the optical microscope when exposed to acidic and alkaline conditions. (see Supplementary Fig. S5). We studied the kinetics of the response of the composite over 24 h and observed that it required an hour to reach and maintain a steady fluorescence state (see Supplementary Fig. S6). We also recorded the autofluorescence behavior of acrylic and found that it was too low to interfere with CsgA–pHuji’s ability to distinguish between acidic and alkaline pH on the textile (see Supplementary Fig. S7).

We also tested the ability of the textile–CsgA–pHuji system to determine the unknown pH of different solutions using the calibration curves seen in Supplementary Fig. S8. As seen in Supplementary Table S1 (Supplementary information) document, when we predicted the pH of unknown solutions with calibration curves made from the same solution, we found the absolute errors to be as low as 0.1–0.65 pH units. When we used the calibration curves obtained from Carmody buffers alone to predict the pH of other solutions, we obtained variations in accuracy ranging from 0.2 pH units to 1.50 pH units. These higher absolute errors are, however, still at par with what is reported for several standard pH-responsive sweat sensors^7,37,38^. Therefore, the protein–textile sensor can be optimally used against a calibration curve with the same composition as the solution of interest. Moreover, the sensitivity of the device is also highest between pH 5.5–8.5 (~ 460 to 830 RFU/pH unit, depending upon the solution used) and reduces considerably above and below this range. This variation in sensitivity also alters the sensor’s ability to predict pH. This sensitivity profile makes the sensor suitable for distinguishing accurately between pH values in its high sensitivity range, and for acting as an ON/OFF sensor for alkaline and acidic environments.

Apart from the stability of the response of the textile–CsgA–pHuji composite over 1 day, we demonstrated the ability of the wearable device to respond to changes in its environment by performing a reversibility assay. We selected two pH buffers that yielded drastically different fluorescence responses (pH 3 and pH 10) and we sequentially immersed the composite sensor in one buffer and the other. We cycled the pH 10 times over 3 days, allowing the sensor to adjust for 1 h prior to recording fluorescence data. Apart from the first data point exposed to pH 10, there was no statistically significant difference between the fluorescence values recorded for the different cycles for the same buffer (Fig. 4c), clearly illustrating the reversibility of the response over days. The reversibility and stability of the fluorescence signal over time opens up exciting avenues for CsgA–pHuji wearable sensors. The textile composites could be adapted for continuous monitoring applications during which changes in the pH of sweat would be detected in real time. The sensor can also provide a binary answer to any wearable applications requiring a visual determination for whether a fluid is alkaline or acidic.

## Conclusion

In conclusion, we have reported a wearable protein-based sensor that can effectively detect pH changes in aqueous solutions, and that could be applied to diagnosing skin conditions via sweat monitoring. To develop this sensor, we demonstrated that genetically programmable curli fibers can be fused with the pHuji protein as a sensing moiety for pH in a quantitative manner. We then integrated these engineered proteins with acrylic samples via a simple and convenient vacuum filtration protocol to create wearable sensors. The resulting materials possess both the fluorescent sensing properties of the proteins, and the mechanical stability and strength of the textile. Using this textile–protein sensing system, we showed that we could distinguish between solutions of varying pH, perform measurements over a few days, and sequentially reuse a single sensor to measure different samples one after the other. The ability of the textile–protein sensors to sustain their steady-state fluorescence behavior over days makes them suitable candidates for continuous monitoring applications. The reversible nature of their responses could further be adapted to creating reusable ON/OFF diagnostic systems. Overall, the bio-derived pH sensor developed here establishes a new field of applications for engineered curli fibers. These composites could be optimized in the future to monitor the pH of various relevant body fluids including sweat from wearable patches or garments, urine from diapers or underwear, and wound exudates from smart bandages. Tailoring curli fibers to act as scaffolds to display various sensing moieties could further allow for introducing enhanced functionalities into the textiles of tomorrow.

## Methods

### Cell strains and plasmids

The pET21d-*csgBACEFG* plasmid and the curli operon deletion mutant strain of *E. coli*, PQN4, were gifts from the Joshi Lab (Harvard University, Boston, MA, USA)^12^. The plasmid we used throughout this paper is an engineered version of pET21d-*csgBACEFG* with a *csgB* deletion in the curli operon, henceforth referred to as pET21d-*csgACEFG.* This single operon after deletion of *csgB* enables the production of curli nanofibers that are no longer adhered to the surface of the bacterium but secreted into the extracellular medium. To design the recombinant gene encoding for a pH-responsive variant of CsgA, pET21d-*csgACEFG* operon was linearized at the C-terminal end of CsgA. A double-stranded DNA fragment comprising a glycine-serine rich flexible linker (F36) and the gene for pHuji were synthesized (Life Technologies) and cloned into the plasmid using isothermal Gibson assembly (New England Biolabs). The plasmid was transformed into MACH1 cells (Life Technologies) and then purified through miniprep (Qiagen). The insertion of the pHuji coding sequence was confirmed through Sanger sequencing (Genome Quebec). The pET21d-*csgACEFG* and pET21d-*csgA*-F36-pHuji-*CEFG* plasmids were transformed into *E. coli* PQN4 to express wild type CsgA (WT CsgA) and CsgA–pHuji fibers respectively. All primer and gene sequences used in this study are described in the Supplementary section. A plasmid containing the *malE* gene (encoding the maltose binding protein MBP), pET21d-MBP was used as negative control for protein expression assays.

### Textiles

A collection of eight woven and non-woven textiles, commonly used in wearable sensors, was sent to us by textile computing company Myant Inc (Ontario). These textiles were categorized on the basis of pore sizes and preliminary indications of mechanical strengths (see Supplementary Fig. S2). Due to its superior tolerance to vacuum filtration, chemical treatments and large pore size in contrast to the other samples, non-woven acrylic was chosen to be the textile candidate of interest for the remainder of the study.

### Production and purification of curli fiber hydrogels

pET21d-*CsgACEFG* and pET21d-*csgA*-F36-pHuji-*CEFG* plasmids were transformed into electrocompetent PQN4 cells and then streaked onto lysogeny broth (LB) agar plates containing 100 µg/mL carbenicillin and 2% glucose (m/v). The plates were incubated at 37 °C overnight. The following day, one colony was picked from each plate and grown overnight in 5 mL LB, 100 µg/mL carbenicillin and 2% glucose at 37 °C and 225 rpm. This small-scale culture was then diluted 100-fold in LB media containing only 100 µg/mL carbenicillin (without glucose) and allowed to express proteins for 24 h at 37 °C and 225 rpm. The cultures were incubated with GdmCl at final concentrations of 0.8 M and stored at 4 °C for 30 min prior to each filtration. GdmCl-treated cultures were passed through polypropylene membranes of pore size 10 µm until the filters were clogged. The filtered biomass was incubated with 5 mL of 8 M GdmCl, followed by vacuum filtration of the liquid and multiple rinses with pure DI water. The nucleic acids in the biomass were treated upon by incubation with 5 mL benzonase nuclease (Sigma-Aldrich, 1.5 U/mL) for 10 min followed by another round of vacuum filtration and extensive water rinses. In order to gelate the protein aggregate and delaminate the resultant curli hydrogel from the filters, they were incubated with 5 mL of 5% SDS (m/v) solution, followed by a final filtration of the residual liquid and extensive DI water rinses. The WT CsgA and CsgA–pHuji hydrogels were gently scraped off the filters and stored at 4 °C until use.

### Curli-textile composite fabrication

The expressed WT CsgA fibers and CsgA–pHuji fibers were integrated with 60 × 40 mm acrylic swatches via vacuum filtration. 45, 60- and 90 mL aliquots of bacterial cultures expressing WT CsgA were incubated with GdmCl at final concentrations of 0.8 M and stored at 4 °C for 30 min prior to each filtration. The cultures were filtered through the textile in the same manner as described in the previous section with an exception of the SDS incubation step. The acrylic–WT curli composites were rinsed well with DI water and stored at room temperature before use. To take into account the reduced yield of CsgA–pHuji in comparison with WT CsgA, we increased the volume of filtered culture to 150 mL for integration of the former with textiles.

### Scanning electron microscopy

For imaging the biofilms, overnight cultures of PQN4 expressing WT CsgA and CsgA–pHuji were filtered onto filter membranes with 0.2 µm pores. To image the acrylic–curli composites, textiles with curli fibers were prepared using the aforementioned vacuum filtration protocol. All the samples to be imaged were fixed with 2% w/v glutaraldehyde and 2% w/v paraformaldehyde for 2 h at room temperature. The samples were then washed with water and treated for fifteen minutes each with increasing concentrations of ethanol (25%, 50%, 75% and 100% v/v). The fixed biomaterials were dried using a critical point dryer prior to sputter coating (Leica ACE600) with platinum to a thickness of 4 nm for 42 s. SEM imaging of the textiles and composites were carried out using a FEI Quanta 450 Environmental

Scanning Electron Microscope (Field Electron and Ion Company).

### Pore size measurements

SEM images of the textiles with the curli fibers were analyzed using the software ImageJ (National Institute of Health). The porosity of the textiles was calculated as the ratio of the percentage surface area of the pores in the threshold image to that of the total area of the image. Surface porosity of textiles with 45, 60 and 90 mL of deposited curli fibers was determined in triplicates. Based on the SEM images, all further acrylic–WT CsgA samples were prepared by filtering 60 mL of culture and acrylic–CsgA pHuji samples with 150 mL of culture.

### Congo Red binding assay

Cell densities of overnight cultures expressing WT CsgA and CsgA–pHuji fibers were measured at 600 nm wavelength using a NanoDrop One UV-Visible Spectrophotometer (Thermo Fisher Scientific). The assay was conducted with amyloid-binding azo dye Congo Red at a concentration of 0.00015% (m/v) using a protocol previously described^20^.

### SDS-PAGE

0.5 mg of lyophilized WT CsgA and CsgA–pHuji fibers were disassembled in hexafluoroisopropanol (HFIP) and trifluoroacetic acid (TFA) at a volume ratio of 1:1.

The solutions were sonicated in an ultrasonic bath sonicator for an hour. The solvents were then allowed to evaporate overnight. The following day, the disassembled proteins were resuspended in 50 µL of sterile DI water and sonicated for 30 min. The samples were electrophoresed in a pre-cast Mini-Protean TGX gel (Bio-Rad Laboratories) at 200 V for 30 min.

### Circular dichroism

1.5 mg/mL of WT and CsgA–pHuji hydrogels in sterile DI water was vortexed until complete dissolution. 200 µL of the sample was then loaded into a circular dichroism (CD) cell and the spectra were read using a Chirascan spectrophotometer (Applied Photophysics). The scans were taken from the wavelength range of 180–260 nm. The CD spectra were normalized using absorbance at 280 nm as a measure of protein concentration.

### Fluorometry of hydrogels and pellets

1.45 mg/mL solution of CsgA–pHuji hydrogel in DI water was vortexed until completely dissolved. 50 µL of the protein solution was treated with 150 µL of a Carmody pH buffer solution, ranging from pH 3–11^39^. Triplicates of each sample were excited at 550 nm and the resultant fluorescence at 598 nm was read using a microplate reader (Tecan Life Sciences). We used the Henderson–Hasselbalch equation (Eq. 4) to obtain the non-linear fit of CsgA–pHuji’s fluorescence behavior. In parallel, concentrated pellets obtained from cultures expressing CsgA–pHuji were treated with 2 mL of the same pH buffers, centrifuged at 4000 × *g* for 5 min and photographed to observe the visual differences in pHuji’s response.

### Tensile strength tests

Tensile strength analysis of the curli–acrylic composites were carried out according to the guidelines mentioned in ASTM D5035-06 (Standard Test Method for Breaking Force and Elongation of Textile Fabrics) with slight modifications to the sample size. Strips of acrylic were integrated with 60 mL of WT CsgA to obtain composites of average densities of 11.5 g m^−2^. Triplicates of each composite were cut into dimensions of 60 × 10 mm (instead of the standard 100 × 150 mm) and plain acrylic (with no curli fibers) was used as the control. Half of the composites were tested dry and the other half were hydrated in water for 1 h to obtain the dry and wet tensile strengths, respectively. Each of these strips was positioned between the parallel plates of the Shimadzu EZ Universal Tensile tester (Shimadzu) with a load cell of 500 N. These strips were stretched until their respective breakage points and the stress–strain curves were plotted.

### Stability tests

Textile squares of 1 cm × 1 cm were cut from acrylic–curli composites and weighed on an ultramicrobalance (Sartorius). Each well in a 12-well plate was filled with one of the following solutions-5% Sodium dodecyl sulphate (w/v), 8 M GdmCl, DI water, 70% ethanol (v/v), buffer of pH 4, buffer of pH 10, artificial sweat and varying concentrations of sodium chloride (1 M–0.001 M).

Solutions of pH 4 and pH 10 were prepared by adjusting the pH of DI water with 1 M HCl and 1 M NaOH, respectively. The composites were incubated with these environmental agents for 10 days with constant agitation. The samples were incubated with DI water to wash off the residual salts and air dried. The weights of the composites after chemical/solvent treatment were taken and loss of curli fiber from the textile substrates were measured using Eq. (1).

1 $$Loss\,of\,curli\,fibers \left( \% \right) = \frac{{W_{i } - W_{f \left( g \right)} }}{{W_{i} \left( g \right)}} \times 100$$

where W_i_ is the initial weight of acrylic–curli composites and W_f_ is the final weight of acrylic–curli composites.

### Water absorption tests

Textile squares of 1 cm × 1 cm were cut from both plain acrylic and acrylic–curli composites. The dry weights of each sample were measured on an ultramicrobalance. These samples were then incubated with 2 mL of DI water for 24 h. The following day, the excess water from the samples were blotted out and the wet weights of each of the samples were measured on the ultramicrobalance.

Water swelling ratio was measured according to Eq. (2).

2 $$Swelling\,ratio{ }\left( {\text{\%}} \right) = { }\frac{{W_{eq} - { }W_{i} { }}}{{W_{i} }} \times 100$$

where W_eq_ is the equilibrium swelling weight and W_i_ is the initial weight of the sample.

### Water vapor transmission tests

Circular swatches of diameter of 1 cm were cut from acrylic and acrylic–curli composites. Glass vials with mouths of diameter of 1 cm were filled with 1 mL of DI water. These circles were capped onto the mouths of these vials and held into place with parafilm surrounding the circumference. The weights of vials were taken and stored at two temperatures—room temperature (20 °C) and human body temperature (37 °C). 10 weight measurements were taken over a span of 24 h. These measurements were used to calculate the water vapor transmission rate using the formula given in Eq. (3).

3 $$WVTR{ }\left( {{\text{g}}\,{\text{m}}^{ - 2} \,{\text{day}}^{ - 1} } \right) = \frac{{{ }W_{i} { }{-}{ }W_{f} { }\left( {\text{g}} \right)}}{{Area{ }\left( {{\text{m}}^{2} } \right) \times { }time{ }\left( {{\text{days}}} \right)}}$$

where WVTR is the water vapor transmission rate of the sample, W_i_ is the initial weight of the glass vial and W_f_ is the final weight.

### Fluorometry of textiles

Cultures of CsgA–pHuji were filtered onto acrylic swatches and samples of 1 mm diameter were cut using a hole punch and placed in the wells of a 96-well plate. The textile samples were incubated with 200 µL of Carmody buffers (pH ranging from pH 4–11) , phosphate-citrate buffers (pH ranging between 3–10) and artificial sweat (Adjusted from pH 4–10 with 1 M HCl and 1 M NaOH) The samples were excited at 550 nm and fluorescence was measured at 598 nm. Measurements were taken every 15 min for 24 h and the responses after an hour were plotted to obtain the standard curve [fit using the Henderson–Hasselbalch equation in Eq. (4)]. After the quantitative analysis, the composite samples were dried and imaged under an optical microscope. To test the reversibility of the sensor, the fluorescence of the composites was measured at buffers of pH 9 for an hour, followed by removal of the samples and reinsertion into wells containing buffers of pH 4 for another hour. This process was repeated for ten cycles and the fluorescence measured at 598 nm. Lastly, the textiles were treated with known buffers like 1× Tris Acetate EDTA, 1× phosphate buffer saline (PBS), artificial sweat, phosphate-citrate buffer and carbonate-bicarbonate buffer. Artificial sweat was prepared by a protocol described previously^40^. The fluorescence intensities of the textile in the presence of these buffers was compared with the standard curve and were used to predict pH^41^. Actual pH of the different buffers were measured using a pH electrode (Sartorius).

4 $$pH{ } = { }pKa{ } + log{ }\frac{{y{ }_{max} - y}}{{y_{min} - y}}$$

where pKa is the dissociation constant, y_max_ is the highest overall fluorescence intensity among all samples, y_min_ is the lowest fluorescence intensity and y is the fluorescence intensity of the sample of interest.

## Supplementary information

Supplementary Information.

## Data Availability

The data that support the findings of this study are available from the corresponding author upon reasonable request.
