# Supplementary material for: Fabrication of fluorescent pH-responsive protein–textile composites
Source: Sci Rep. 2020 Aug 3;10:13052. doi: 10.1038/s41598-020-70079-x (PMC7400762; doi:10.1038/s41598-020-70079-x)
Supplement: Supplementary file 1 — Supplementary Information. [file 41598_2020_70079_MOESM1_ESM.docx]

**Supplementary Information**

Fabrication of Fluorescent pH-Responsive Protein-Textile Composites

*Dalia Jane Saldanha, Zahra Abdali, Daniel Modafferi, Bita Janfeshan and Noémie-Manuelle Dorval Courchesne**

# Isolation of curli fiber-based hydrogels via vacuum filtration

We differentiated between WT CsgA (14.7 kDa) and CsgA-pHuji (40.3 kDa) on the basis of their molecular weights and the absence of a WT CsgA monomer band in the CsgA-pHuji lane, and the presence of a thick band around 40 kDa corresponding to the fusion protein. Non-specific bands seen in the WT CsgA lane in the range of 40-50 kDa could either be impurities from *E. coli*’s proteome ^[1]^ or dimers and trimers of WT-CsgA subunits that tend to reassemble quickly in aqueous buffers. Similarly, bands showing between 75-250 kDa could correspond to oligomers of CsgA-pHuji that re-aggregated upon resuspension of the disassembled proteins in loading buffer.

**Figure S1**. SDS-PAGE of disassembled CsgA-pHuji (left lane) and WT CsgA (right lane) fibers. The complete length of all three consecutive lanes are visible in this image. We uniformly enhanced the brightness and contrast of the entire image by 20% to better visualize the bands. In the left lane, the box corresponds to the CsgA-pHuji monomer of molecular weight 40.3 kDa. In the right lane, the box corresponds to the WT CsgA monomer of molecular weight 14.7 kDa.

# Selection of candidates for textile-protein fabrication

A collection of textiles was obtained from Myant Inc. for entrapment of curli fibers via vacuum filtration (**Figure S2a**). SEM images of the textiles revealed that all the textiles differed from each other in terms of pore size ranging from as high as 500 µm (e.g. Acrylic) to as low as 0.1 µm (e.g. PAN). The fabrics with small or negligible pores like PAN and nylon-6 also prevented the passage of bacterial cells during filtration, thereby leading to widespread contamination of the composite, as seen in **Figure S2b**. Electrospun fabrics like PVR, PVDF, PVB and PAN were also unable to sustain the stresses of filtration, often either deforming or tearing after the entire process, making them ineffective candidates for textile-curli composites (**Figure S2c and S2d**).

**Figure S2.** a) Different textiles provided by Myant Inc. (Ontario) for fabrication of textile-protein composites. Top left to right: Acrylic, Cellulose Acetate (CA), Nylon-6, Silver-coated Nylon (Ag-Nylon). Bottom left to right: Polyvinyl Butyral (PVB), Polyvinylidene difluoride (PVDF), Polyvinyl Rayon (PVR) and Polyacrylonitrile (PAN). Scale bars: 500 µm b) Trapping of dense bacterial populations in textiles with low porosities like PAN and Nylon-6. c) Deformation of PAN after filtration with WT CsgA culture. d) Disintegration of textiles like cellulose acetate and PAN after vacuum filtration.

**Characterization of acrylic and CsgA-pHuji composites**

Dry composites of acrylic and CsgA-pHuji prepared by vacuum filtration showed a widespread distribution of the latter across the textile matrix. These composites also demonstrated stress-strain curves similar to composites of acrylic and WT-CsgA, indicating that variants of curli fibers can easily integrated with textiles without hampering the overall mechanical behavior.

**Figure S3.** Stress strain curve of acrylic and CsgA-pHuji composite in the dried state. Inset: SEM image of the composite prepared by filtering 90 mL of CsgA-pHuji producing bacterial culture onto acrylic. Scale bar: 500 µm.

# Time-based analysis of the fluorescence of CsgA-pHuji hydrogels

In order to determine the longevity of CsgA-pHuji’s response, fluorescence properties of the responsive protein were recorded every fifteen minutes for 18 hours (**Figure S5**). It was noted that while the CsgA-pHuji responds instantaneously to solutions of different pHs and even at time 0 generates a characteristic Michaelis-Menten trend,^[2]^ the fluorescence intensity under each condition stabilizes only after one hour. A possible explanation could be the reaggregation of CsgA-pHuji hydrogel in water-based solvents over time. CsgA-pHuji bearing an amyloid framework does not completely dissolve in most solvents. Interaction of curli fibers with water might cause them to reaggregate and separate out of the pH buffer after one hour. This could result in exposing the pHuji’s fluorophore groups and concentrating the signal.

**Figure S4.** pH-dependent fluorescence response of CsgA-pHuji hydrogels recorded over a period of 18 hours.

**Visualization of acrylic-curli composites at different pHs**

Just like our analysis for protein hydrogels in **Figure 3g**, **(See main article)** we first visually observed subtle color changes in textile-curli composites as a function of pH to evaluate the functionality of the sensor. Under optical microscope, the composites with entrapped CsgA-pHuji fibers were darker when incubated in buffers at alkaline pHs compared with acidic pHs. Note that proteins cover cm-sized textile pieces, with an average constant protein density across samples, but slight inhomogeneity at the hundreds of micron level.

**Figure S5**. Optical microscope images of acrylic-CsgA-pHuji composites incubated at different pHs. Scale bars: 500 µm.

# Time-based analysis of the fluorescence of acrylic-CsgA-pHuji composites

The fluorescence intensity of CsgA-pHuji was also measured for long timeframes when integrated with acrylic. Similar to the hydrogel, the curli-textile composites begin to exhibit steady fluorescence intensities only at the 1-hour time point. However, in contrast to the pure hydrogels, there is a dip in fluorescence intensity to reach steady state.

**Figure S6.** pH-dependent fluorescence response of Acrylic-CsgA-pHuji hydrogels recorded over a period of 22 hours.

# Autofluorescence of non-woven acrylic

When we incubated acrylic-only samples with buffers of different pH and monitored their fluorescence emissions upon excitation at 550 nm, autofluorescence was detected (**Figure S7**). The autofluorescence of acrylic, however, is 5-fold lesser than the composites under acidic conditions and 20-fold lesser under alkaline conditions. This eliminates chances of the textile itself interfering with the sensitivity of the sensor.

**Figure S7**. Autofluorescence of acrylic controls incubated with varying pH buffers for one hour.

**Prediction of pH using the curli-based sensor**

A non-linear fit of CsgA-pHuji’s fluorescence behavior on the textile was carried out using the Henderson-Hasselbalch equation. We used this fitted curve, generated in **Figure 4b (See main article)** to confirm our ability to predict the pH of test solutions using this textile-curli sensor. We selected five test solutions (buffers with different compositions, and artificial sweat), and measured the fluorescence emission values of the sensor when incubated in their presence. We then predicted the pH values of the test solutions within a 95% confidence interval. The calibration curves we obtained from Carmody buffers, artificial sweat and phosphate-citrate buffers for the prediction test and their associated confidence intervals are seen in **Figure S8**. We compared these predictions with the pH values obtained for the test solutions with a standard pH meter (**Table S1**). We found that choice of calibration curve played an important role in the accuracy of our sensor’s pH prediction. When we referred to calibration curves obtained from the same solution, we were able to predict unknown pHs with absolute errors ranging from 0.1-0.3 pH units for buffered solutions and 0.65 pH units for artificial sweat. When we predicted the pH using the calibration curves from Carmody buffers, we observed larger variations in accuracy ranging from 0.2-1.5 pH units. However, even these higher error ranges agree with trends reported in literature. ^[3-5]^ The errors associated with testing the pH of some buffers were higher than the others implying the varying effects that different ions can have on the dielectric environment of the fluorophore, causing a shift in its pKa. Ions could also adversely affect the excited state proton transfer required for emission of red light, destabilizing the anionic chromophore and thereby affecting slightly the accuracy of the sensor. It is also important to note that the sensitivity of the device is highest between the pH ranges of 5.5 and 8.5 (depending upon the solution in use). However, for physiological parameters like skin pH, for which relative ranges (rather than exact values) are sufficient to make an informed diagnosis, this textile-protein sensor provides an effective medium to understand skin health.

**Figure S8**. Calibration curves (solid lines) for Carmody buffer (CB), Artificial sweat (AS) and Phosphate-Citrate buffer (PC) for the pH prediction test with associated 95% confidence intervals (dotted lines).

**Table S1.** **Prediction of pH by acrylic-curli composite**. Measured pH is pH predicted using the textile-composite sensor of different buffers. 95% confidence intervals for the measured pH is shown. Actual pH is the pH of the buffer measured using a standard pH electrode. Relative errors represent the percentage difference between the measured pH and actual pH of each buffer. Reference curves refer to the calibration curve used to predict unknown pH. Variation from actual pH represents the absolute error associated with the prediction, in pH units.

| **Sample** | **Measured pH** | | **95% CI** | **Actual pH**  **(± 0.01)** | **Relative**  **Error (%)** | **Reference curve** | **Variation from actual pH (pH units)** |
| --- | --- | --- | --- | --- | --- | --- | --- |
| Carmody buffer - I | 7.20 | 7.20-7.37 | | 7.50 | 3.9 | Carmody | 0.30 |
| Artificial sweat - I | 7.01 | 7.01928-7.01929 | | 6.36 | 10.3 | Artificial sweat | 0.65 |
| Phosphate- Citrate- I | 7.27 | 7.2712795  -7.2712797 | | 7.40 | 1.7 | Phosphate-Citrate | 0.13 |
| Artificial sweat - II | 7.48 | 7.46 to 7.51 | | 6.48 | 15.58 | Carmody | 1 |
| Carbonate-Bicarbonate | 8.31 | 8.24 to 8.40 | | 9.46 | 12.08 | Carmody | 1.15 |
| Phosphate-Citrate - II | 5.82 | 5.60 to 5.97 | | 5.63 | 3.44 | Carmody | 0.19 |
| Tris Acetate EDTA | 6.46 | 6.41 to 6.51 | | 7.97 | 18.88 | Carmody | 1.51 |
| Phosphate Buffer Saline | 6.61 | 6.57 to 6.64 | | 7.19 | 8.05 | Carmody | 0.58 |

**Table S2:** Primers and gene fragments used in this study.

| **Name of construct** | **Sequence** |
| --- | --- |
| Forward primer (C-terminal end of CsgA) | 5’ GTACTGATGAGCGGTCGCGTTGTTA 3’ |
| Reverse primer (C- terminal end of CsgA) | 5’CTAATACATCATTTGTATTACAGAAACAGGGCGCAAG 3’ |
| Flexible GS linker | 5’GGTGGATCTGGTAGCAGTGGCTCTGGTGGATCAGGGGGTGGAAG TGGCTCCTCTGGGAGCGGGGGTTCGGGAGGTGGCTCGGGTTCATC TGGTAGTGGCGGTTCGGGT 3’ |
| pHuji | 5’ATGGTTTCTAAAGGGGAGGAAAATAATATGGCTATTATCAAAGAAT TTATGCGTTTCAAGGTGCATATGGAAGGGTCTGTCAATGGACATGA  ATTTGAAATCGAGGGTGAAGGTGAGGGGCGTCCTTACGAAGCCTTT  CAGACTGCGAAACTGAAGGTGACTAAAGGCGGACCGCTGCCGTTT  GCCTGGGACATTTTGTCTCCGCAGTTCATGTACGGATCTAAGGTCT  ATATTAAACACCCTGCTGACATCCCGGACTACTTCAAACTTTCTTTT  CCGGAGGGTTTTCGTTGGGAACGTGTCATGAATTTCGAAGATGGGG  GAATTATTCACGTTAACCAAGACTCTAGTCTGCAAGATGGGGTGTTC  ATTTACAAGGTCAAATTACGCGGCACTAACTTTCCCAGCGATGGCC  CTGTAATGCAGAAGAAGACGATGGGGTGGGAGGCATCTGAAGAAC  GTATGTACCCTGAGGACGGGGCATTGAAATCTGAAATCAAATACCG  TTTGAAGTTAAAGGACGGAGGACATTACGCAGCCGAGGTGAAAACA  ACTTACAAAGCCAAGAAACCAGTCCAGTTGCCTGGTGCGTACATCG  TTGATATTAAGCTGGACATTGTCTCGCATAACGAAGATTACACGATT  GTTGAACAGTATGAGCGTGCTGAGGGTCGTCATTCCACGGGAGGC ATGGATGAATTATACAAATAA 3’ |

**REFERENCES**

1. Dorval Courchesne, N-M., Duraj-Thatte, A., Tay, P. K. R., Nguyen, P. Q. & Joshi, N.S. Scalable production of genetically engineered nanofibrous macroscopic materials via filtration. *ACS Biomaterials Science & Engineering* **3**, 733-741 (2016).
2. Shen, Y., Rosendale, M., Campbell, R. E. & Perrais, D. pHuji, a pH-sensitive red fluorescent protein for imaging of exo-and endocytosis. *J Cell Biol* **207**, 419-432 (2014).
3. Bandodkar, A. J. et al. Tattoo-based potentiometric ion-selective sensors for epidermal pH monitoring. Analyst 138, 123-128 (2013).
4. Caldara, M., Colleoni, C., Guido, E., Re, V. & Rosace, G. Optical monitoring of sweat pH by a textile fabric wearable sensor based on covalently bonded litmus-3-glycidoxypropyltrimethoxysilane coating. Sensors and Actuators B: Chemical 222, 213-220 (2016).
5. Curto, V. F. *et al.* Real-time sweat pH monitoring based on a wearable chemical barcode micro-fluidic platform incorporating ionic liquids. *Sensors and Actuators B: Chemical* **171**, 1327-1334 (2012).
